# A *PTPN11* mutation in a woman with Noonan syndrome and protein-losing enteropathy

**DOI:** 10.1186/s12876-020-01187-1

**Published:** 2020-02-13

**Authors:** Na Wang, Wen Shi, Yang Jiao

**Affiliations:** 1 Department of Pulmonary and Critical Care Medicine, Peking Union Medical College Hospital, Chinese Academy of Medical Sciences & Peking Union Medical College, Beijing, 100730 China; 2 Department of Gastroenterology, Peking Union Medical College Hospital, Chinese Academy of Medical Sciences & Peking Union Medical College, Beijing, 100730 China; 3 Department of General Internal Medicine, Peking Union Medical College Hospital, Chinese Academy of Medical Sciences & Peking Union Medical College, No. 1, Shuaifuyuan, Wangfujing St. Beijing, Beijing, 100730 China

**Keywords:** Noonan syndrome, Protein-losing enteropathy, *PTPN11*, Hypoproteinemia

## Abstract

**Background:**

Noonan syndrome is an autosomal dominant, variably expressed multisystem disorder characterized by specific facial and cardiac defects, delayed growth, ectodermal abnormalities, and lymphatic dysplasias. Lymphedema and chylous pleural effusions are common in Noonan syndrome, but protein-losing enteropathy (PLE) has only rarely been described in the condition and little is known about its genetic associations.

**Case presentation:**

We report the case of a 30-year-old Chinese woman who developed severe recurrent edema and hypoproteinemia. Gastroduodenoscopy showed a “snowflake” appearance of lymphangiectasia in the duodenum, and CT reconstruction of the small intestine showed segmental thickening of the intestinal wall with localized stenosis. Whole exome sequencing revealed that the patient harbored a pathogenic variant of *PTPN11* (c.A922G p.N308D), which was unfortunately inherited by her 2.5-year-old daughter who had short stature and atrial septal defect but no hypoproteinemia.

**Conclusions:**

This case of Noonan syndrome with PLE was associated with a *PTPN11* mutation. A comprehensive review of PLE in Noonan syndrome revealed that PLE often presents late in this context but there is no clear genotype-phenotype correlation. Genetic evaluation with next-generation sequencing can be useful for securing the diagnosis and planning early intervention and management.

## Background

Noonan syndrome (MIM: 163950) is an autosomal dominant, variably expressed multisystem disorder characterized by specific facies, cardiac defects, delayed growth, auditory deficits, ectodermal abnormalities, and lymphatic dysplasias (< 20%) [1]. While lymphedema and chylous pleural effusions are common in Noonan syndrome [2], protein-losing enteropathy (PLE) has rarely been reported and little is known about its genetic causes or associations in this context [3, 4]. PLE is usually syndromic or associated with non-syndromic primary intestinal lymphangiectasia. On its own, PLE has been reported in association with specific genetic mutations including in *CCEB1*, *FAT4* [5], *PIEZO1* [6], *FOXC2* [7], *CD55* [8], and *DAGT* [9].

Recent high-throughput genetic analyses with genome-wide association studies (GWAS) and whole exome sequencing (WES) have revealed a number of genetic variations that contribute to the susceptibility of Noonan syndrome [1]. All the genes currently implicated in Noonan syndrome encode proteins integral to the RAS–MAPK pathway, an important signal transduction pathway controlling cellular proliferation, differentiation, survival, and metabolism, with specific disease-causing mutations usually determining the Noonan sub-phenotype. In particular, patients carrying variants of *PTPN11* (roughly 50%), an upstream member of the MAPK signaling cascade, tend to have pulmonary stenosis, short stature, lower IGF1 concentrations [10], bleeding diatheses, and juvenile myelomonocytic leukemia [11]. Patients harboring *RAF1* (roughly 10%) variants in serine 259 and serine 621 have hypertrophic cardiomyopathy [12, 13], and those with *KRAS* (< 2%) variants have delayed cognitive development [14] and intellectual disability [15]. Patients with *SOS1* variants (~ 10%) have a higher prevalence of ectodermal abnormalities [16] and are taller than average [17], and those with *NRAS* mutations account for < 2% of cases and currently do not have a discernible genotype-phenotype correlation [18]. However, the association between PLE and specific genetic mutations in Noonan syndrome has yet to be determined.

Here we present the case of a 30-year-old Chinese woman presenting with recurrent edema and hypoproteinemia. Using WES and Sanger sequencing, we discovered that the patient carried the common pathogenic *PTPN11* variant (c.A922G p.N308D) of Noonan syndrome. Unfortunately, screening of family members revealed the same mutation in her two and a half-year-old daughter. Her daughter had a relatively mild phenotype with facial dysmorphia and short stature. This case provides the opportunity to review the clinical features and genetics of PLE in Noonan syndrome and highlights the importance of mutation testing, genetic counselling, and family member screening to provide early intervention.

## Case presentation

A 30-year-old woman was admitted to hospital with progressive lower limb edema over 8 months and occasional convulsions. She had initially ignored the bilateral lower limb edema but, as the edema gradually expanded to the abdomen, upper limbs, and even head and face, she was admitted to the local hospital for treatment of hypoproteinemia (albumin 21 g/L; normal range 35–52 g/L) and hypocalcemia (calcium 1.93 mmol/L; 2.13–2.70 mmol/L). However, no diagnosis was made, and she was eventually referred to the tertiary hospital. She reported a past medical history of tetralogy of Fallot at 7 years of age, for which she underwent surgery at age 14. Her menstrual cycle was normal, and she had given birth to a daughter. Her child, who was two and a half years old at presentation, was born normal but had a history of feeding difficulties and atrial septal defect. There was no other family history of note.

Upon admission, the patient was conscious and her vital signs were within normal limits. She was 150.1 cm tall and weighed 55 kg, and her daughter showed short stature (height 132.5 cm, − 3.6SD). They shared the same dysmorphic facies with hypertelorism, low-set ears, and a posterior hairline. Limb circumferences were 24.5 and 25 cm at 10 cm above the upper edge of the patella and 14 and 13.5 cm at 10 cm below the edge of the patella. In addition, early grade 3 diastolic murmurs were audible over the pulmonary and tricuspid valves.

Thorough biochemical screening was performed. Unsurprisingly, many nutritional indices were reduced beyond the lower limit of normal values except for liver function and renal function. The lymphocyte count was 0.29 × 10^9^/L (0.80–4.00 × 10^9^/L); hemoglobin 108 g/L (110–150 g/L); total protein 31 g/L (60–85 g/L); albumin 19 g/L (35–52 g/L); calcium 1.33 mmol/L (corrected calcium 1.77 mmol/L; 2.13–2.70 mmol/L). All immunoglobulins were decreased.

**Fig. 1:**
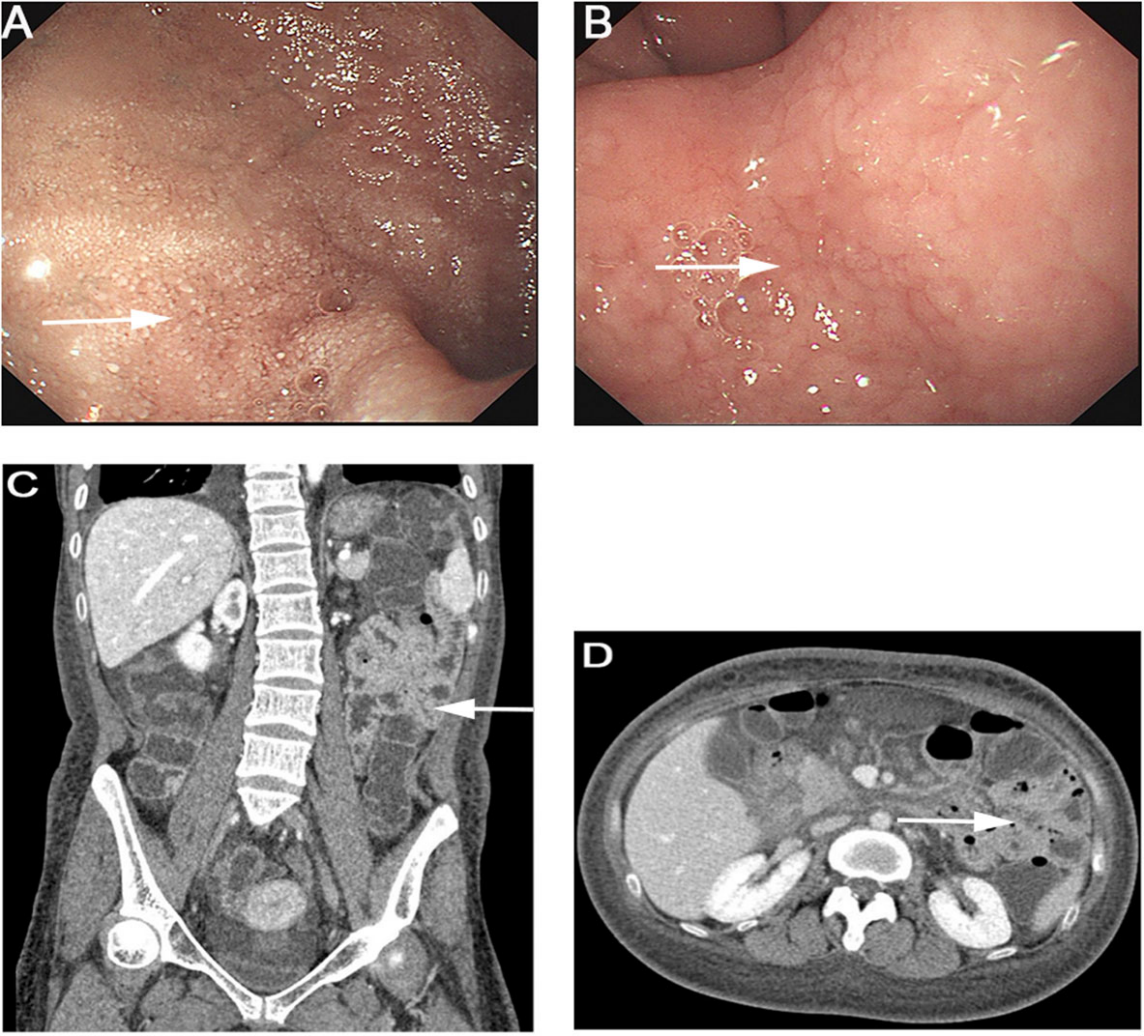
**a** shows the snowflake appearance of the duodenum and **b** shows the granuloid changes in the gastric antral mucosa by electronic gastroscopy. Reconstructive CT of the small intestine in (**c** & **d**) demonstrate segmental thickening of the intestinal wall with local intestinal stenosis. Arrows highlight the indicated features

In view of the definite diagnosis of hypoproteinemia, the digestive, endocrine, and cardiac systems were next screened in detail. For the digestive system, a stool occult blood test was positive and the D-xylose absorption test was 0.9 g/5 h (normal > 1.2). Gastroduodenoscopy showed a snowflake appearance in the duodenum (Fig. 1a and b), a sign of lymphangiectasia. CT reconstruction of the small intestine showed that the descending duodenum wall was coarsely thickened and the small intestinal wall was sectionally thickened, enhanced, and locally narrowed (Fig. 1c and d). There was no obvious colonic abnormality.

Lymphatic imaging of the lower limbs showed lymphangiectasis and bilateral widening of the venous angle in the mediastinum. Imaging at 1.5 h showed diffuse radioactive uptake in the small intestine, which diminished by 5 hours but at which time showed new areas of radioactive uptake in the ascending colon. Whole body lymphatic imaging indicated widening of the lymphatics in both lower limbs and a flaky radioactive enhancement shadow was seen in the abdominal cavity within 3 h. By 6 h, the hepatic flexure and transverse colon could be visualized.

In the light of paroxysmal tetany, endocrine system screening mainly focused on metabolic indicators. Parathyroid hormone levels were 122.20 pg/ml (12.0–68.0 pg/ml), synchronous blood calcium was 1.55 mmol/L, synchronous albumin was 19 g/L, synchronous 24 h urine calcium was 0.10 mmol/24 h, total 24-hydroxyvitamin D was < 3.00 ng/ml (8.0–50.0 ng/ml), 1,25-dihydroxyvitamin D3 was 24.42 pg/ml (19.6–54.3 pg/ml), blood magnesium was 0.45 mmol/L (0.70–1.10 mmol/L), and β-collagen degradation product was 1.23 ng/ml (0.21–0.44 ng/ml). Echocardiography revealed no abnormality in cardiac structure or function except for changes associated with the previous repair.

**Fig. 2:**
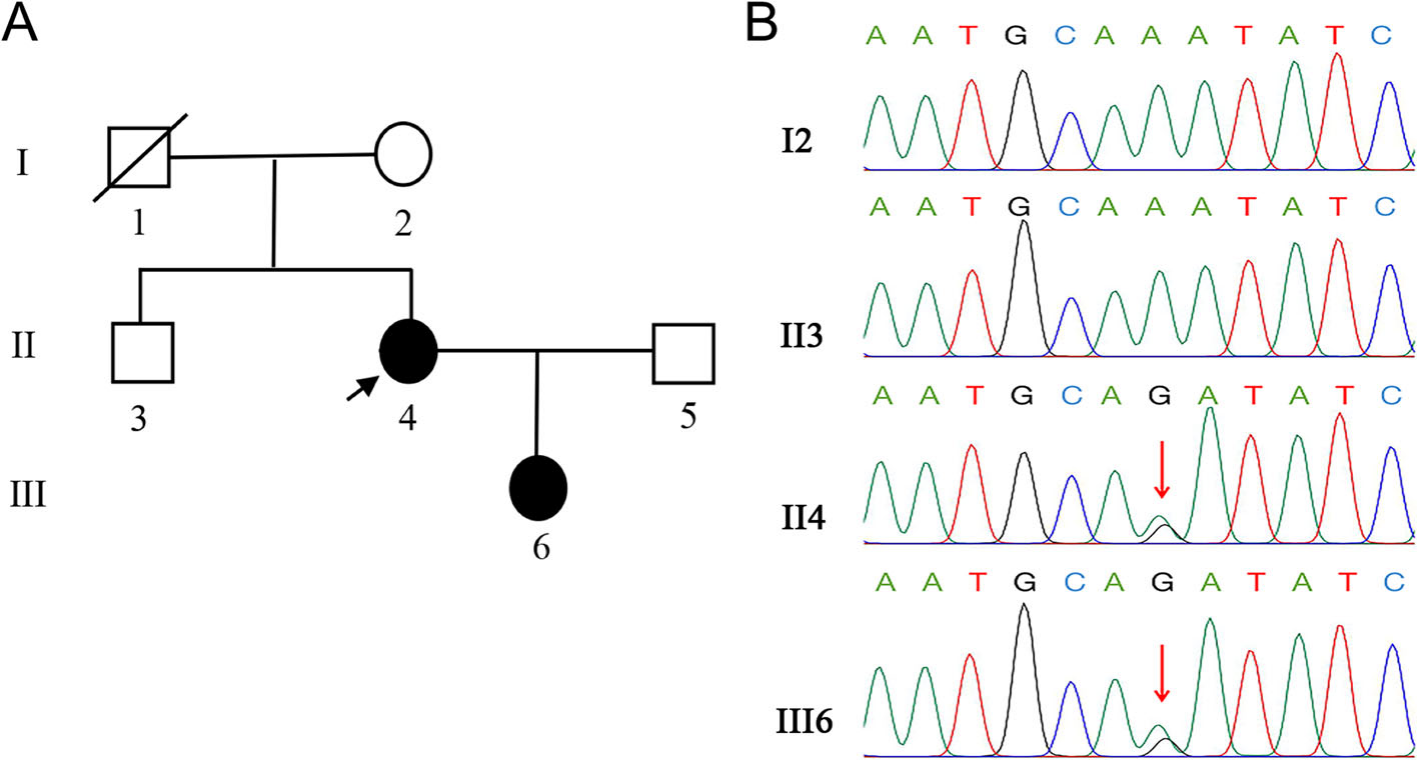
**a** shows the pedigree of the patient’s family. Patients are represented in black and the arrow represents the proband, which is the patient discussed in this article. **b** shows the Sanger sequencing of the PTPN11 gene in the family. A missense mutation was found in PTPN11 (c.A922G p.N308D) of the patient (II4), which was inherited by her daughter (III6). The patient’s father died of acute cerebrovascular disease before genetic testing

The diagnosis remained uncertain, so the patient and family agreed to whole exome sequencing. A pathogenic variant in *PTPN11* (c.A922G p.N308D) was detected and confirmed by Sanger sequencing, which also revealed the same mutation in the patient’s daughter (Fig. 2a & b). No mutation was detected in the patient’s mother, and the father had died some years before from cardiovascular disease.

The patient was prescribed a medium-chain triglyceride diet. Example dietary changes included the use of 3–4 g/day coconut oil for cooking rather than the intake of long-chain fats; increased intake of high-quality proteins like egg white, skimmed milk, whey protein, and lean meat; and avoidance of crude fiber (e.g., grains, celery) and high-fat food (e.g., cream, fatty pork). In the following 8 months, there were no further episodes of edema or convulsions with periodic infusion of albumin and oral calcium intake. Her daughter was short and met the criteria for taking growth hormone replacement. Regular follow-up of the daughter was also advised.

## Discussion and conclusions

Here we describe a patient with severe edema and tetany developing over a long timeframe. There was no obvious cause for her diffuse lymphangiectasia, but clinical observation revealed abnormal facies and she had a history of congenital heart disease, raising suspicion of a congenital abnormality. However, next-generation sequencing was required to confirm the presence of a pathogenic *PTPN11* mutation to explain congenital heart disease and PLE. The genotype-phenotype correlation of Noonan syndrome and PLE has not been established. The occurrence or severity of lymphatic abnormality might differ according to the specific genetic mutation, but data on this hypothesis is lacking.

A comprehensive literature search of the PubMed and CNKI databases from 1972 to 2019 using the search terms “Noonan syndrome, protein-losing enteropathy” and “Noonan syndrome, PTPN11” revealed only nine reported cases (Table 1). Male and female patients with Noonan syndrome who developed PLE were similarly affected. However, patients usually developed PLE after Noonan syndrome was diagnosed (16.4 ± 7.9 vs. 7.3 ± 7.1 years; *p* = 0.03), and our patient’s daughter will require long-term follow-up to anticipate the development of this complication. All patients had congenital heart disease, two of whom had tetralogy of Fallot, a rare cardiac abnormality [4]. Other common manifestations were edema (6/8, 75%) and hypoalbuminemia (8/8; total protein 33.6 ± 7.9 g/L; albumin 18.9 ± 3.6 g/L). Other isolated clinical manifestations included hepatomegaly [21], intractable bleeding from cutaneous lymphatic malformations [25], drug reactions with eosinophilia and systemic symptoms (DRESS), and thrombotic microangiopathy [26]. Our patient demonstrated occasional tetany due to hypocalcemia, which was treatable with calcium supplements. In the published cases, two patients died of severe complications: one of heart failure and another of hemorrhagic pancreatitis after a valvuloplasty.

**Table 1:** Summary of all patients with Noonan syndrome-associated PLE identified in the literature (1972–2019)

Cases	Sex	The onset of NS (yr)	The onset of PLE (yr)	Symptoms	Cardiac disorder	TP (g/L)	Alb (g/L)	Transnodal lymphangiography	Treatments	Follow-up
Matsumoto et al. [19]	F	17	17	No obvious clinical symptoms	HCM	31	15	Absent thoracic duct abdominal collateral lymphatics and bilateral iliac lymphangiectasia	Steroid therapy (1 mg/kg/d) Low-fat, protein-rich diet supplemented with medium-chain triglycerides	Relieved
Mizuochi et al. [20]	F	1.5	8	Edema, abdominal pain, diarrhea	ASD PVS	32	18		Spironolactone (2.5 mg/kg/d) Furosemide (2.0 mg/kg/d)	Relieved
Keberle et al. [4]	M	6	13	Edema of abdomen and hydrocele testis	ASD PVS	32	18	Protein loss from the small intestine	Albumin (2.5 g) Growth hormone	Relieved
Keberle et al. [4]	M	19	21	Tibial edema Clubbing	Fallot’s tetralogy	41	26	Intestinal protein loss predominantly in the ileum	Low-fat, protein-rich diet, medium-chain triglycerides	Relieved
O’Sullivan et al. [21]	M	7	22	Diarrhea	PVS	< 20			Anti-heart failure	Died^a^
Herzog et al. [22]	F	0.9	15	Ankle swelling	ASD PVS	45		Hypoplasia of the lymphatics of the extremity and multiple ectatic lymph vessels in the mediastinal area and right supraclavicular area	Medium-chain triglyceride diet	Relieved
Vallet et al. [23]	M	0.3	6	Diarrhea Anasarca, chylorrhea from the inguinal skin	PVS	38	20	Unavailable	Medium-chain trigIycerides and a low-fat diet	Died^b^
Joyce et al. [24]	F	Unavailable	27	Bilateral lower limb and genital swelling	PVS	Unavailable	Unavailable	Lymph reflux/rerouting. R: popliteal LN present. Contrast in vulva and multiple channels in both legs	Low-fat MCT diet	Relieved
Joyce et al. [24]	M	Unavailable	55	Bilateral lower limb and suprapubic swelling	ASD	Unavailable	Unavailable	Unavailable	Unavailable	Unavailable
Our case	F	7	30	Extrimitis edema	Fallot’s tetralogy	31	19	Lymphangiectasis and bilateral widening of the venous angle in the mediastinum and small intestine	Low-fat, medium-chain triglycerides	Relieved

Abbreviations: *PLE* Protein-losing enteropathy, *HCM* Hypertrophic cardiomyopathy, *ASD* Atrial septal defect, *PVS* Pulmonary valve stenosis, *TP* Total protein, *Alb* Albumin; *a* means the patient died of heart failure; *b*, the autopsy revealed the immediate cause of death to be hemorrhagic pancreatitis after a valvuloplasty for PVS. The normal range for TP and Alb is 60-85 g/L and 35-52 g/L, respectively

*PTPN11* encodes the protein tyrosine phosphatase SHP-2, which has an amino N-SH2 domain and a phosphotyrosine phosphatase domain (PTP) to switch the protein between its inactive and active conformations [1]. The N-SH2 domain plays a key role in maintaining inactive SHP-2 [27], with the N-SH2 and PTP domains sharing a broad interaction surface. Several hydrogen bonds between the N-SH2/PTP domains form the most critical catalytic sites. Alterations in these critical amino acids might disturb the equilibrium between active and inactive forms of SHP-2 [28]. Indeed, the G > C point mutation at position 417 (Glu139Asp) is the only mutation identified which alters an amino acid in the C-SH2 domain, which contributes to substrate specificity and binding affinity [28].

**Fig. 3:**
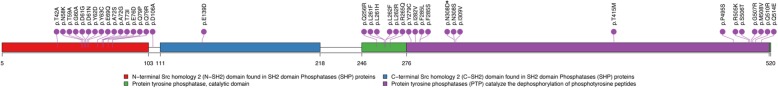
Distribution of the missense mutations identified in PTPN11, as provided by the UniProt database (uniprot.org). The mutation detected in our patient is marked with an asterisk. The different colored rectangles represent the protein domains and the purple spheres represent amino acid changes at different mutation sites

An energetics-based structural analysis indicated that a gain-of-function mutation in *PTPN11* could be responsible for the disease [29]. There are 40 reported *PTPN11* mutations (UniProt.org; Fig. 3), and several large retrospective studies have indicated that different *PTPN11* mutations are correlated with some sub-phenotypes. Musante et al. [28] screened for mutations in *PTPN11* in 96 familial and sporadic cases and found that the phenotypes associated with *PTPN11* mutations included (from the most to least common) dysmorphic features (hypertelorism, low-set ears, down-slanting palpebral fissures), cryptorchidism, short stature, cardiac defects, and myelodysplasia. In another large retrospective study [29], the variant was found to be more frequent in familial cases than sporadic cases. Genotype-phenotype correlation analysis revealed that pulmonary stenosis was more prevalent in subjects with Noonan syndrome with *PTPN11* mutations than those without (70.6% vs. 46.2%; *p* < 0.01). Furthermore, a pathogenic *PTPN11* mutation was predicted to confer a 3.5-fold increased risk of developing cancer compared with the general population [30]. In a Japanese study of 41 Noonan syndrome patients, mutations at codons 61, 71, 72, and 76 were frequently identified in patients with leukemia, including those with JMML, MDS, AML, and ALL [31]. In terms of other disease associations, three different *PTPN11* mutations (E69K, T507K, and Y62C) were identified in 89 primary neuroblastomas [32] and, in a case report, a patient with Noonan syndrome caused by a germline mutation in exon 13 of *PTPN11* (c1507G > C, p.Gly503Arg) developed Hodgkin’s lymphoma [33], which was also associated with congenital refractory chylothorax and subcutaneous edema [34].

With respect to *PTPN11* mutations in Noonan syndrome, Joyce et al. [24] identified *PTPN11* (c.181G > A, p.Asp61Asn) and *PTPN11* (c.188A > G, p.Tyr63Cys) mutations in two Noonan syndrome patients with PLE. Interestingly, Noonan syndrome and cardiofaciocutaneous syndrome (CFC) are both RASopathies that share some similarities including the same genetic variants [35]. Joyce et al. [24] also reported three patients with CFC-PLE carrying *KRAS* (c.178G > C, p.Gly60Arg), *BRAF* (c.770A > G, p.Gln257Arg), and *RIT1* (c.246 T > G, p.Phe82Leu) mutations. Whether the *PTPN11* mutation or the N308D variant is causal for PLE remains to be determined, but other lymphatic disorders have been reported in association with *PTPN11* mutations, including jugular lymphatic obstruction with a heterozygous T > C change in exon 8 [36] and lymphatic dysplasia in the lung and mesentery with a heterozygous mutation at G503R [37]. Interestingly, the N308D mutation is frequently hereditary rather than sporadic. Tartaglia et al. [38] reported an A → G transition at position 922 in exon 8 in three families, as did Musante et al. [28] in another family. Our case further contributes to the evidence that this mutation clusters in families. While PLE has no clear genotype-phenotype correlation, there are certainly several cases suggesting that these mutations may be pathogenic; further work is needed in larger cohorts.

In our case, the patient carried the *PTPN11* variant, which was inherited by her daughter. It was unclear whether this variant was inherited or sporadic (Fig. 3), although her father, who had died of acute cerebrovascular disease aged 55 years and could therefore not be tested, showed no clinical manifestations of Noonan syndrome.

There is no standard treatment for PLE in Noonan syndrome. Most patients reported in the literature recovered after treatment, which included periodic supplemental albumin and long-term medium-chain triglycerides. Moreover, glucocorticoids and diuretics achieved long-term symptomatic relief in some patients with Noonan syndrome [19, 20]. Systemic corticosteroids such as prednisone have been used for their anti-inflammatory effects [39]. Diuretics may decrease the CVP, which promotes lymphangiogenesis and lymphangiectasia [40]. Early growth hormone replacement in children can result in near adult heights later in life [41].

PLE is often congenital in etiology and associated with Hennekam syndrome (HS), Turner syndrome (TS), and Noonan syndrome (Table 2). HS is a recessive disorder that can have disordered small intestinal lymphangiogenesis associated with mutations in *CCBE1* and *FAT4* [42, 43]. TS is an allosomal disorder in which infants with the 45,X karyotype are most likely to have congenital lymphedema [44].

**Table 2:** Differential characteristics of three congenital diseases associated with PLE

Disease	Genetic property	Causative genes	Morbidity	Pattern	Height (cm)	
					M	F
Hennekam syndrome	Recessive inheritance	*CCEB1, FAT4, ADAMTS3*	Rare< 50 cases/worldwide	Lymphangiogenesis can occur in many areas, the most common being the small intestine but also the kidney, chest, pericardium, thyroid gland and skin [39].	156.3 ± 11.3	155.3 ± 4.7
Turner syndrome	Allosomal inheritance	45, X 46, X, i (Xq) Mosaicism	1/1500–2500	Infants with a 45,X karyotype are the most likely to have congenital lymphedema [40]		141.3 ± 5.6
Noonan syndrome	Autosomal dominant	*PTPN11, SOS1, RAF1, KRAS, SHOC2, NRAS*	1/1000–1/2500	Lymphangiogenesis restricted to pterygium and limbal lymphedema and often combined with cardiac disease.	157.3 ± 7.4	146.8 ± 6.9

Abbreviation: *PLE* Protein-losing enteropathy

In conclusion, here we report a case of Noonan syndrome with PLE carrying a *PTPN11* variant. PLE occurs late in patients with Noonan syndrome. Noonan syndrome may be confused with other genetic diseases clinically, and genetic evaluation with next-generation sequencing to identify the genetic basis can be helpful. Finally, screening family members, especially children, may provide the definitive diagnosis to guide early intervention.

## Data Availability

Data sharing is not applicable to this article as no datasets were generated or analyzed during the current study.

## Abbreviations

CT: Computed tomography
DRESS: Drug reactions with eosinophilia and systemic symptoms
GWAS: Genome-wide association studies
HS: Hennekam syndrome
MAPK: Mitogen activated protein kinase
PLE: Protein-losing enteropathy
TS: Turner syndrome
WES: Whole exome sequencing
